# The Dual Role of Amyloid Beta-Peptide in Oxidative Stress and Inflammation: Unveiling Their Connections in Alzheimer’s Disease Etiopathology

**DOI:** 10.3390/antiox13101208

**Published:** 2024-10-08

**Authors:** Hugo Fanlo-Ucar, Pol Picón-Pagès, Víctor Herrera-Fernández, Gerard ILL-Raga, Francisco J. Muñoz

**Affiliations:** 1 Laboratory of Molecular Physiology, Department of Medicine and Life Sciences, Faculty of Medicine and Life Sciences, Universitat Pompeu Fabra, 08003 Barcelona, Spain; hugo.fanlo@upf.edu (H.F.-U.); ppicon@ibecbarcelona.eu (P.P.-P.); victor.herrera@upf.edu (V.H.-F.); gerard.ill@upf.edu (G.I.-R.); 2 Laboratory of Molecular and Cellular Neurobiotechnology, Institute of Bioengineering of Catalonia (IBEC), 08028 Barcelona, Spain; 3 Centro de Investigación Biomédica en Red sobre Enfermedades Neurodegenerativas (CIBERNED), 08028 Barcelona, Spain

**Keywords:** Alzheimer’s disease, amyloid β-peptide, neurodegeneration, nitro-oxidative stress, BACE1

## Abstract

Alzheimer’s disease (AD) is a progressive neurodegenerative disease, and it is currently the seventh leading cause of death worldwide. It is characterized by the extracellular aggregation of the amyloid β-peptide (Aβ) into oligomers and fibrils that cause synaptotoxicity and neuronal death. Aβ exhibits a dual role in promoting oxidative stress and inflammation. This review aims to unravel the intricate connection between these processes and their contribution to AD progression. The review delves into oxidative stress in AD, focusing on the involvement of metals, mitochondrial dysfunction, and biomolecule oxidation. The distinct yet overlapping concept of nitro-oxidative stress is also discussed, detailing the roles of nitric oxide, mitochondrial perturbations, and their cumulative impact on Aβ production and neurotoxicity. Inflammation is examined through astroglia and microglia function, elucidating their response to Aβ and their contribution to oxidative stress within the AD brain. The blood–brain barrier and oligodendrocytes are also considered in the context of AD pathophysiology. We also review current diagnostic methodologies and emerging therapeutic strategies aimed at mitigating oxidative stress and inflammation, thereby offering potential treatments for halting or slowing AD progression. This comprehensive synthesis underscores the pivotal role of Aβ in bridging oxidative stress and inflammation, advancing our understanding of AD and informing future research and treatment paradigms.

## 1. Introduction

Alzheimer’s disease (AD) is a devastating condition that has reached pandemic proportions, with 55 million people affected in 2019 [1] and projections estimating that this number will rise to 139 million by 2050 [2].

AD diagnosis is mainly made by cognitive tests, brain imaging, and genetic analysis. It is classified into two main types: sporadic and familial. Sporadic AD is the most common form, accounting for most cases, and typically occurs in individuals after the age of 65 without a clear genetic link. Its onset is influenced by a combination of genetic, environmental, and lifestyle factors. Familial AD, on the other hand, is rarer and occurs in families with a history of the disease, often appearing at an earlier age (30s or 40s). This form is associated with specific genetic mutations, which will be discussed in this review.

There are no effective treatments for AD, despite the amyloid β-peptide being identified more than 40 years ago [3]. By the end of the 20th century, AD was characterized histopathologically and molecularly as a brain disease marked by extracellular senile plaques, composed mainly of aggregated Aβ, and intracellular neurofibrillary tangles made of tau.

The primary component of senile plaques is a peptide, consisting of 40 to 42 amino acids, termed amyloid-β [3] (from the Greek term *amylon*, meaning starch) due to its propensity to adopt a β-sheet conformation and aggregate into unbranched, twisted fibrils that have a starch-like affinity for iodine [4]. Similar characteristics are observed in various pathological systemic amyloids, such as transthyretin [5], amylin [5], or ABri and ADan peptides [6,7]. Regarding Aβ, it produces brain damage, increasing oxidative stress and inflammation, which are the main topics of this review.

## 2. Aβ Production

The amyloid precursor protein (APP) is an integral type I transmembrane glycoprotein present in most human cells. It has three major isoforms, with the largest one, containing 770 amino acids, being the most abundant in the brain [8]. In humans, APP coexists with other APP-like proteins, APLP1 and APLP2, which do not contain the Aβ sequence and appeared earlier in vertebrate evolution. These proteins are reported to regulate cell adhesion [8], among other functions. Specifically, APP has been implicated in synaptogenesis [9], axonal transport [10], and the regulation of the GABA receptor [11].

**Figure 1:**
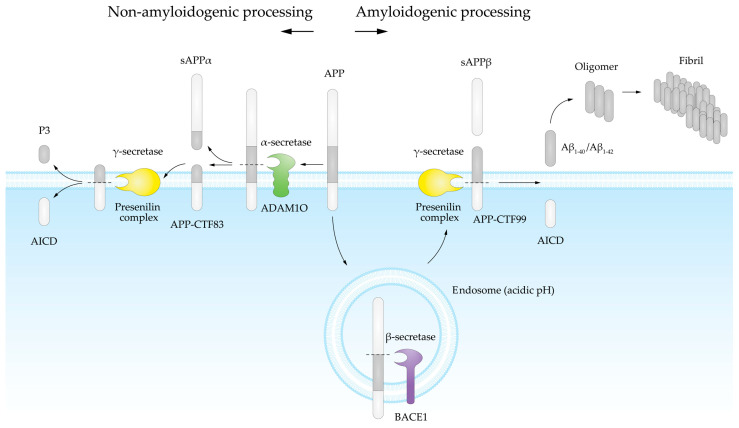
Physiological APP cleavage pathways are depicted. The non-amyloidogenic pathway is shown on the left side of the figure. In this pathway, α-secretase cleaves APP, producing sAPPα and CTF83. Subsequently, CTF83 is cleaved by γ-secretase, releasing the P3 peptide extracellularly and AICD intracellularly. The amyloidogenic pathway is shown on the right side of the figure. This pathway involves β-secretase cleavage, which takes place mainly within the intracellular endosome pathway, thus producing sAPPβ and CTF99. CTF99 is then cleaved by γ-secretase at the cell membrane, releasing AICD intracellularly and Aβ extracellularly.

Aβ is produced through the enzymatic processing of APP [12,13] (Figure 1). When APP is cleaved by an α-secretase, Aβ is not produced, as this enzymatic activity cuts APP in the middle of the Aβ sequence (Figure 1). However, when APP is sequentially cleaved by a β-secretase and a γ-secretase, Aβ is released [14].

The amyloidogenic pathway begins with the cleavage of APP by β-secretase, specifically the β-site APP cleaving enzyme 1 (BACE1), a transmembrane aspartyl protease enzyme [15,16]. BACE1 has a homolog, BACE2, which exhibits minimal β-secretase activity and primarily functions outside the brain, playing roles in pigmentation and glucose homeostasis [17,18,19]. The β-secretase activity occurs mainly within the intracellular endosome pathway, where the acidic pH activates BACE1 [20,21,22]. Soluble APP-β (sAPPβ) is a byproduct of BACE1 cleavage, which does not induce neural progenitor cell proliferation [23].

Subsequently, the heterogeneous presenilin complex, consisting of presenilin (PSEN), nicastrin (NCT), presenilin enhancer 2 (PEN-2), and anterior pharynx defective 1 (APH1) [24,25,26], executes γ-secretase activity at the plasma membrane, releasing the Aβ fragment and the intracellular fragment APP intracellular domain (AICD). This process is similar to Notch proteolytical cleavage, in which the functional form of Notch is cleaved by the γ-secretase complex, releasing the intracellular fragment Notch intracellular domain (NICD). The fact that NICD has transcriptional regulatory activity in the nucleus [27] has prompted researchers to investigate the potential transcriptional function of AICD [28].

The variability in Aβ length is due to γ-secretase activity, with Aβ_1-40_ being the most common form and Aβ_1-42_ occurring to a lesser extent [29]. There are also forms ranging from 38 to 43 amino acids. The human Aβ_1-43_ amino acid sequence is:

DAEFR^5^HDSGY^10^EVHHQ^15^KLVFF^20^AEDVG^25^SNKSA^30^IIGLM^35^VGGVV^40^ IA^42^T^43^,
with the last three amino acids being critical for its aggregation tendency. Certain mutations also play a significant role in Aβ aggregation, mainly those located around the amino acid at position 20 [29,30,31,32,33].

**Figure 2:**
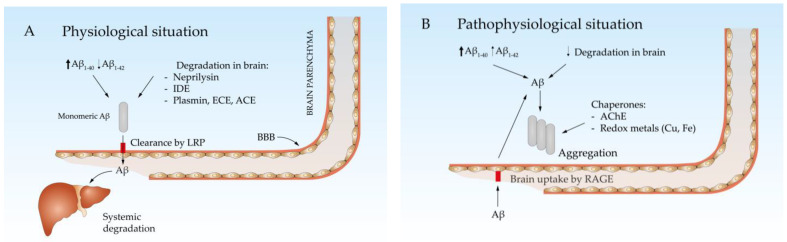
Physiological (**A**) and pathophysiological (**B**) brain Aβ equilibrium. (**A**) Aβ is predominantly produced as Aβ_1-40_ and can be degraded within the brain parenchyma or cleared to the blood via LRP, ultimately being degraded in the liver. (**B**) With age, the production of Aβ_1-42_ increases, while its degradation within the brain and clearance decrease. This leads to aggregation, facilitated by protein chaperones and redox-active metals.

Both APP cleavage pathways occur physiologically from childhood onward. Aβ produced by various tissues circulates in the blood and is degraded mainly by the liver [34,35,36] (Figure 2A). In the brain, Aβ can be degraded by the insulin-degrading enzyme (IDE) [37], neprilysin (NEP) [38], and other enzymes such as plasmin [39], endothelin-converting enzyme (ECE) [40], or angiotensin-converting enzyme (ACE) [41]. The remaining Aβ is released into the blood through the blood–brain barrier (BBB) [42,43]. Aβ is transported from the cerebrospinal fluid (CSF) to the bloodstream via low-density lipoprotein receptor-related protein 1 (LRP1)- or very low-density lipoprotein receptor (VLDLR)-mediated transcytosis [44,45]. Both are membrane receptors that facilitate the movement of Aβ across the BBB by binding to it and transporting it through cells (transcytosis). This process helps clear Aβ from the brain, playing a critical role in maintaining brain homeostasis and preventing its accumulation.

With age, the concentration of Aβ in the brain increases due to various mechanisms. Aβ production is enhanced because of increased translation and activity of BACE1 [46] and presenilin [47] driven by nitro-oxidative stress, which is strongly associated with aging [48]. Additionally, the expression of NEP and IDE decreases with age [49,50] and LRPs become less efficient at removing Aβ [51]. In this scenario, the BBB acts as a dam that retains Aβ in the brain, leading to increased concentrations and favoring Aβ aggregation (Figure 2B). While monomeric Aβ is not only not neurotoxic but in fact has been proposed to play some physiological roles, such as regulating insulin signaling [52], its β-sheet conformation allows it to form oligomers and fibrils that are neurotoxic to both neurons and vascular cells in the brain [53,54,55]. Some extracellular molecules, such as transthyretin, clusterin (also termed Apolipoprotein J), and albumin, act as chaperones by binding monomeric Aβ and preventing its aggregation [56,57,58]. However, their function is impaired by certain polymorphisms, as seen with clusterin, where specific variants are considered risk factors for late-onset AD [59].

## 3. Synaptotoxicity and Neuronal Death

Histopathological and experimental evidence shows that oxidative stress [60,61,62,63] and inflammation [64,65] are directly involved in Aβ synaptoxicity and neuronal death. They will be discussed later in this review. It is also known that Aβ oligomers, given their small size and their hydrophobic nature due to their secondary structure of β-sheets, can interact with membrane proteins present in the synaptic cleft, altering their function.

**Figure 3:**
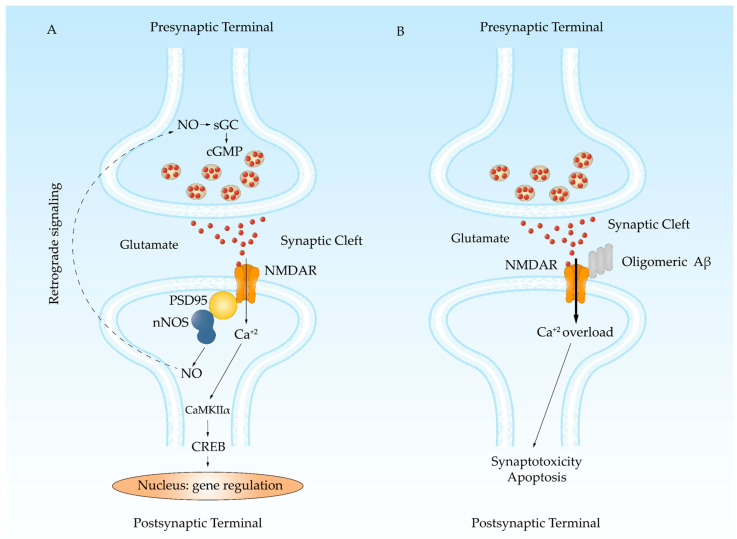
NMDAR is a target for Aβ oligomers. (**A**) LTP allows memory formation by the continuous stimulation of the glutamatergic signaling. Glutamate increases calcium entrance activating nNOS. NO induces the release of glutamate by the presynaptic terminal. Calcium also activates CaMKIIα, that phosphorylates CREB, triggering the transcription of genes needed for synaptic spine growth. (**B**) Aβ oligomers bind to NMDAR impairing a proper closing, which produces a leak of calcium into the cell that induces synaptotoxicity and neuronal death.

Among the different proteins with which oligomeric Aβ can interact, the NMDA receptor (NMDAR) stands out (Figure 3). It has been demonstrated in cultures of hippocampal neurons that stimulation with NMDA following oligomeric Aβ treatment prolongs the channel open state. This effect was not observed when cells were treated with Aβ oligomers and a high potassium solution, which activates voltage-dependent calcium channels [51]. This suggests that the oligomeric Aβ effect is specific to NMDAR activation, although it may not be entirely exclusive. Ultimately, Aβ oligomers cause greater calcium influx, which affects the reservoirs responsible for calcium homeostasis. It promotes ryanodine receptor (RyR)-mediated calcium release from the endoplasmic reticulum and mitochondrial calcium uptake [66], leading to mitochondrial dysfunction and reactive oxygen species (ROS) production [67,68]. All these processes generate an environment of low amplitude and long-lasting abnormal calcium signaling that promotes synaptotoxicity [69,70,71].

Other proteins susceptible to the Aβ effects are the α-7-nicotinic receptors, which present high Aβ affinity, and become activated at picomolar concentrations of Aβ but moderately inhibited at higher concentrations [72,73]. This is supposed to contribute to the cholinergic deficit characteristic of AD [74,75], and the rationale basis for the use of anticholinesterase drugs that can increase the bioavailability of acetylcholine in the synaptic cleft. The degeneration of the cholinergic pathways proposed to be initiated in the nucleus basalis of Meynert (or nucleus basalis magnocellularis) could also be contributing significantly to the cholinergic deficit in AD [74,75].

Other mechanisms of Aβ toxicity have been proposed, such as the regulation of intracellular calcium by SURF4 throughout the store-operated calcium channel impairment [76] or the insertion of oligomers into membranes forming ion-permeable pores [77]. Nonetheless, it is difficult to consider that the possible formation of a pore has a relevant contribution to the general neuronal damage, since pores appear to be quite scarce, and dimers, which have been demonstrated to be highly toxic, cannot produce a transmembrane pore [78].

Finally, GSK-3β, a serine/threonine kinase, contributes to neuronal death in AD. GSK-3β has been extensively studied in the context of AD, and it has been implicated in a variety of cellular processes that are disturbed in AD, including Aβ generation [79], tau phosphorylation [80], synaptic plasticity [81], and inflammation [82]. Furthermore, brain GSK-3β levels increase with age [83], and it is found hyperactive in AD samples [84].

## 4. Oxidative Stress in AD

Oxidative stress occurs when there is an excess in the production of ROS, surpassing the antioxidant defenses. ROS are highly reactive molecules containing oxygen, including free radicals like superoxide (O₂^−^) and hydroxyl (OH·), and non-radicals like hydrogen peroxide (H₂O₂) [85].

**Figure 4:**
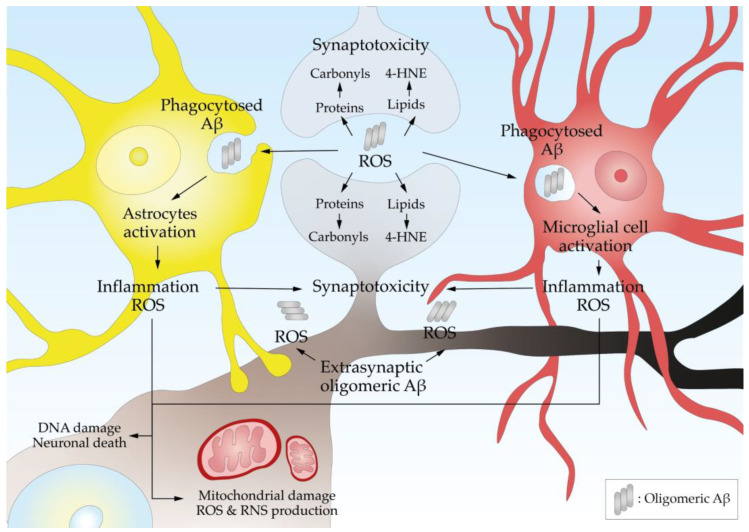
Aβ oligomers produce oxidative stress and neuroinflammation. Synaptic and extrasynaptic Aβ oligomers produce ROS that damage proteins, lipids, and DNA. The Aβ oligomers attract astrocytes that phagocytose them, which triggers their activation, releasing proinflammatory factors and more ROS. Microglia are attracted by chemokines and, after activation, also release proinflammatory factors. All together, these processes produce synaptotoxicity and neurotoxicity.

ROS damage DNA, proteins, and lipids, leading to synaptotoxicity and neuronal death (Figure 4). A marker of oxidative DNA damage, 8-Hydroxy-2′-Deoxyguanosine (8-OHdG), is elevated in the brains of AD patients [86] and reflects the extent of oxidative damage to genetic material. Malondialdehyde (MDA) and 4-hydroxynonenal (4-HNE) are highly reactive byproducts of lipid peroxidation, whose adducts are found at higher levels in AD brains [87,88]. The oxidation of proteins at various residues forming carbonyls has been extensively documented [89,90].

The brain is particularly susceptible to oxidative damage due to its high oxygen consumption [91], abundant lipid content [92], and relatively low antioxidant defenses [93]. Elevated levels of oxidative markers have been found in AD patients [94], suggesting a tight link between oxidative stress and disease progression but also that oxidative stress contributed to the onset of the disease [95,96].

The primary sources of oxidative stress in the brain include mitochondrial respiration and enzymatic reactions involving oxidases, peroxidases and oxygenases. Their activities are increased in AD [97]. Furthermore, Aβ oligomers contribute to oxidative stress by generating ROS [98,99]. The aggregation of tau into paired helical filaments (PHFs) can impair cellular processes and exacerbate oxidative damage, further promoting neuronal dysfunction and death [100].

Finally, homocysteine, an amino acid that is an intermediate product in the metabolism of methionine, can lead to increased oxidative stress and inflammation [101], both of which are critical factors in the development and progression of AD. Homocysteine has been proposed to exacerbate the formation of Aβ plaques [102]. It can also induce apoptosis and excitotoxicity in neurons [103] and endothelial dysfunction [104] (further contributing to cognitive decline).

### 4.1. Metals and Aβ

Aggregated Aβ generates H_2_O_2_ and OH-· through the reduction of metal ions such as iron and copper [98,99,105]. These metals are highly concentrated in Aβ deposits and have been proposed as factors contributing to AD etiology due to their redox activity [106]. Copper and iron can catalyze the production of ROS through Fenton reactions, leading to oxidative stress and neuronal damage [107,108,109,110]. Zinc, while essential for brain function, can induce the aggregation of Aβ peptides by stabilizing their oligomeric forms [111]. The imbalance of these metals disrupts cellular processes, increases ROS production, and exacerbates the neuroinflammatory response.

In particular, copper imbalance has been closely linked to AD as it results from a shift in metal ion pools within the brain [112]. Normally, tightly bound copper ions play crucial roles in energy production and antioxidant defense. However, as copper becomes increasingly loosely bound, it exacerbates oxidative stress [112]. This transition, which may be worsened by aging processes, can disrupt mitochondrial function, deplete energy reserves in neurons with high metabolic demands, and lead to enhanced protein misfolding and aggregation [94].

### 4.2. Mitochondrial Dysfunction in AD

The mitochondria, as the powerhouse of the cell, are the main site for ROS production during ATP synthesis. Aβ-induced ROS lead to mitochondrial dysfunction mainly due to the inhibition of the electron transport chain [113]. Mitochondrial dysfunction is evident in AD, impairing oxidative phosphorylation [114], increasing ROS production [115], reducing ATP levels [116], and impairing mitochondrial dynamics [117]. This dysfunction contributes to neuronal injury and cognitive impairment. The accumulation of oxidative damage within mitochondria further impairs their function, creating a detrimental feedback loop that accelerates the progression of AD [118]. The oxidative damages affect to mitochondrial proteins [119]. Aconitase, which is part of the tricarboxylic acid (TCA) cycle, exhibits high levels of oxidation in AD [120]. It contains an iron-sulphur cluster that is particularly vulnerable to ROS. Oxidation of aconitase disrupts the TCA cycle, leading to reduced energy production and increased mitochondrial dysfunction [120]. VDAC, part of the mitochondrial permeability transition pore, has been also reported to be oxidized, affecting ionic homeostasis and contributing to apoptosis [121].

Mitochondrial DNA (mtDNA) encodes essential mitochondrial proteins, and it is oxidized in AD [122]. ROS-induced damage to mtDNA results in mutations and impaired synthesis of critical ETC components, further exacerbating mitochondrial dysfunction [122,123].

### 4.3. Oxidation of Biomolecules

Oxidative stress leads to damage to neuronal lipids, proteins, and DNA, contributing to neuronal apoptosis and synaptic dysfunction. Furthermore, ROS and reactive nitrogen species (RNS) stress can activate inflammatory pathways and exacerbate neuroinflammation [124]. The combined effects of oxidative stress, nitro-oxidative stress, and neuroinflammation contribute to the cognitive decline and memory impairment in AD patients due to the damage to neuronal structures and synaptic connections [125].

Oxidative stress causes significant damage to DNA, including base modifications, strand breaks, and the formation of DNA–protein cross-links [126]. 8-OHdG is found at elevated levels in the brains of AD patients [86]. In neurons, which have limited capacity for DNA repair [127,128], such damage can accumulate over time along the life of the individuals and trigger apoptotic pathways [129] and inflammatory responses [130], further promoting neurodegeneration.

Oxidative modification of proteins forms carbonyls, compromising protein physiological functions [131,132], as seen in the former paragraphs. It can also result in the formation of advanced glycation end-products (AGEs), impairing protein function and contributing to cellular dysfunction [133,134,135,136,137,138]. Furthermore, ROS can induce reversible oxidation of thiol groups in sulphur-containing residues (cysteine or methionine). It induces a wide range of oxidative post-translational modifications, including sulfenic, sulfinic, and sulfonic acids, and crosslinking through the formation of disulphide bonds. A particular case of crosslinking is S-glutathionylation, in which sulphur-containing residues bind glutathione (GSH) [139]. However, under high levels of ROS, some of these post-translational modifications can turn irreversible and disrupt protein physiological functions [140]. In fact, AD patients present higher levels of disulphide bond-induced protein crosslinking compared to healthy individuals [141].

In the brain, which is rich in polyunsaturated fatty acids, lipid peroxidation produces major damages in the neuronal membrane. MDA and 4-HNE are highly reactive and can form adducts with proteins and nucleic acids [142,143]. In AD, increased levels of lipid peroxidation have been observed, correlating with the severity of the disease [143]. Lipid peroxidation can impair membrane-bound receptor functioning and enzymes, disrupting neuronal signaling and promoting synaptic dysfunction and neuronal loss [144].

As mentioned before, Aβ can be oxidized in the presence of copper [99], which increases its misfolding into Aβ sheets, forming toxic oligomers. Interestingly, oxidation of methionine 35 has been shown to be a retardant of Aβ aggregation [145]. Protein tau has also been reported to be oxidized [146], contributing to its misfolding [147].

In transgenic mouse models of AD, oxidative stress markers begin to appear at different stages depending on the model. In 5xFAD mice, known for their rapid disease progression, oxidative stress markers appear around 3 to 6 months of age, leading to early and pronounced oxidative damage, characterized by the increased presence of biomarkers such as 8-OHdG in plasma and MDA in brain and liver [148]. In Tg2576 and APP/PS1 mice, oxidative stress markers typically become detectable around 6 months of age [149,150,151], with increased levels as the disease progresses. In all of the models, between 6 and 12 months, higher levels of oxidative stress are generally observed, reflecting increased damage to lipids, proteins, and DNA. Both astrocytes and microglia show increased activation during this period, contributing to elevated oxidative stress [152,153,154]. In the advanced stages (12 to 18 months and beyond), oxidative stress markers are typically at their highest levels, correlating with advanced neurodegeneration and extensive plaque deposition [155,156,157].

### 4.4. Oxidative Stress and Aβ Production

Oxidative stress contributes to the production [46,95,96] and aggregation of Aβ [158]. Furthermore, Aβ itself can generate ROS [98], creating a vicious cycle of oxidative damage and Aβ production. ROS can promote the amyloidogenic pathway by increasing the activity of BACE1 by activating stress-activated protein kinases, such as c-Jun N-terminal kinase (JNK) and p38 mitogen-activated protein kinase (p38-MAPK), which induce the transcription of BACE1 [95,96]. Furthermore, the translation of BACE1 mRNA is dependent on the activation of the kinases of the translation factor eIF2. These kinases phosphorylate eIF2 at its α subunit when challenged with stressful stimuli such as oxidative stress [46], nitric oxide (NO) production [159], or virus infection such as the one produced by the herpes simplex virus 1 (HSV-1) [160].

Oxidative stress can also impair the clearance of Aβ by affecting the function of proteolytic enzymes and the ubiquitin-proteasome system [161], increasing its concentration inside the brain, and contributing significantly to AD onset and progression.

## 5. Nitro-Oxidative Stress in AD

Nitro-oxidative stress is driven by ONOO-, which is not a free radical but a highly reactive anion formed by the reaction of NO with the superoxide anion [162]. Its reactivity leads to the nitration of biomolecules, mainly some amino acids such as tyrosines, producing nitrotyrosines [163]. This is a pathological post-translational modification that alters and potentially suppresses the biological function of a protein [47,162,164,165]. On the other hand, nitrosative processes are produced by NO directly and are commonly physiological posttranslational modifications, in many cases reversible [162].

### 5.1. NO and Its Functions

NOSs, which exist in several forms, are not considered isoforms since they are encoded by different genes [162]. They catalyze the production of NO from L-arginine and they play distinct roles in biological systems. The types of NOS are endothelial NOS (eNOS), neuronal NOS (nNOS), and inducible NOS (iNOS). eNOS is a constitutive enzyme of the endothelial cells, where it plays a key role in regulating vascular tone, blood pressure, and preventing platelet aggregation, but it is also expressed in other cell types like neurons and glia [162,166]. nNOS is a constitutive enzyme expressed in neurons and involved in neurotransmission, playing a key role in brain function, including memory and learning [162,166]. nNOS is also expressed in other cell types [162,166]. iNOS is produced by immune cells and glial cells in response to inflammatory stimuli and is part of the immune defense mechanisms, helping to destroy pathogens [162,166], but potentially contributing to tissue damage if overproduced. This dual behavior is evident since genetic deletion of iNOS in a mouse model of AD promotes neurodegeneration [167], and iNOS inhibition could be an effective approach in treating AD and other neurodegenerative diseases [168].

### 5.2. Mitochondria and Nitro-Oxidative Stress

Mitochondrial NOS (mtNOS) is an isoform of nNOS [163] expressed in the matrix and the inner membrane of mitochondria. It regulates mitochondrial respiration by modulating the electron transport chain and ATP production by inhibiting cytochrome c oxidase [169]. NO from mtNOS also influences mitochondrial biogenesis and dynamics [117]. Dysregulation of mtNOS activity can lead to mitochondrial dysfunction, contributing to neurodegenerative diseases [170] and cardiovascular diseases [171].

### 5.3. Nitrosylation and Biomolecules

Nitrosylation is a post-translational modification that involves the covalent attachment of NO to organic molecules without altering the substrate charge, leading to the formation of C-nitroso, N-nitroso, O-nitroso, or S-nitroso derivatives [171]. Most commonly, it binds to the thiol group of cysteine residues on proteins, modulating their function. This process, known as S-nitrosylation, is reversible and plays a regulatory role in cellular signaling [172]. S-nitrosylation regulates the physiological function of numerous proteins [163].

In a pathological scenario, inflammatory factors (TNF-α, IL-1β, IL-6) contribute to an increased production of NO mainly by the activation of the expression of iNOS [173], leading to excessive S-nitrosylation [174]. In AD, this modification becomes dysregulated and impairs the function of proteins involved in synaptic function, such as NMDAR [175], and mitochondrial dynamics, such as dynamin-related protein 1 (Drp1) [176], or even can induce apoptosis by the S-nitrosylation of the caspase cascade [177].

### 5.4. Nitration and Biomolecules

RNS can modify DNA, proteins, and lipids [162]. 8-Nitroguanine (8-NO2G) reflects the nitration of guanine residues in DNA and is indicative of nitrative stress and DNA damage in AD [178]. The nitration of tyrosine residues, forming 3-nitrotyrosine, is a marker of nitrotyrosination in AD [133,179]. These modifications alter the structure and function of proteins, impairing their normal activity.

The nitrotyrosination of the glycolytic enzyme triosephosphate isomerase induced by Aβ decreases the glycolytic flow [133]. Moreover, it triggers the production of the highly neurotoxic methylglyoxal [133,135], which glycates proteins [138]. Nitrative stress has also been implicated in the formation of neurofibrillary tangles [133,180,181].

Aβ can undergo nitration at the tyrosine in position 10, which enhances its misfolding into Aβ sheets, leading to the formation of toxic oligomers and stabilizing them [182].

RNS also induces the peroxidation and the nitration of lipids, altering the integrity of the membrane and the trigger of intracellular signaling [183,184].

### 5.5. Nitro-Oxidative Stress and Aβ Production

The nitrotyrosination of presenilin 1 (PS1), the catalytic subunit of γ-secretase, increases the association of the PS1 fragments, PS1-CTF and PS1-NTF, which form the active catalytic center of the γ-secretase complex [47]. Peroxynitrite also shifts Aβ production towards Aβ_1-42_ and increases the Aβ_1-42_/Aβ_1-40_ ratio [47], the pathophysiological situation found in AD patients.

## 6. Neuroinflammation in AD

Inflammaging refers to the chronic, low-grade inflammation that typically accompanies aging, contributing to the development of age-related diseases such as cardiovascular disease, diabetes, and neurodegenerative disorders [185]. This persistent inflammatory state is believed to result from the cumulative effect of lifelong exposure to various stressors, such as infections, environmental toxins, and lifestyle factors, which gradually impair the immune system’s regulatory functions. Key mechanisms underlying inflammaging include cellular senescence, the production of proinflammatory cytokines, and alterations in the gut microbiome [186]. AD is included in the concept of inflammaging since the major risk factor for AD is aging, it is tightly linked to cardiovascular diseases and diabetes, and there are inflammatory processes in the brain [187].

The accumulation of Aβ triggers an inflammatory response, leading to neuronal loss and synaptic dysfunction that contribute to the cognitive deficits observed in AD patients [188]. The inflammatory response is carried out by astrocytes and microglial cells [189], which migrate toward the Aβ deposits and phagocyte the oligomers and fibrils, producing proinflammatory cytokines, the activation of the complement system in the brains of AD patients, and the production of ROS [190] (Figure 4).

### 6.1. Astroglia in AD

Astrocytes are essential for maintaining the homeostasis of the central nervous system (CNS). They are star-shaped cells with numerous branching processes that extend throughout the brain and spinal cord. They are classified into two main types: fibrous astrocytes, predominantly found in white matter, and protoplasmic astrocytes, found in grey matter [191]. This morphological diversity allows astrocytes to interact with neurons, blood vessels, and other glial cells, facilitating their functions.

#### 6.1.1. Astrocytic Functions

They support neurons by regulating the extracellular environment, maintaining the BBB, modulating synaptic activity, and responding to injury or disease [192]. They also maintain the ionic and chemical balance [192]. Astrocytes provide metabolic support to neurons by supplying essential nutrients, such as glucose and lactate [193], and also lipidic molecules, such as cholesterol, through ApoE-containing lipoproteins [194,195]. They regulate cerebral blood flow through their interactions with blood vessels [196], ensuring an adequate supply of oxygen and nutrients to active brain regions.

Additionally, astrocytes uptake and recycle neurotransmitters, particularly glutamate [197], which is vital for preventing excitotoxicity and maintaining synaptic health. They release gliotransmitters too, such as the neurodepressor ATP and the NMDAR modulator D-serine, which influence synaptic activity and plasticity [198,199]. Through their interactions with synapses, astrocytes contribute to processes like long-term potentiation (LTP) and long-term depression (LTD) [200], essential for learning and memory.

#### 6.1.2. Aβ and Astrocytes

Triggered by neuronal injury and the presence of Aβ deposits, reactive astrocytes exhibit increased expression of glial fibrillary acidic protein (GFAP) [201,202] and an alteration in calcium regulation [203], resulting in metabolic dysfunction [204,205]. It impairs astrocytes’ ability to regulate neurotransmitters, mainly glutamate, through the glutamate transporter 1 (GLT-1), which increases extracellular glutamate and causes excitotoxicity [206].

In relation to oxidative stress, reactive astrocytes release ROS in response to aggregated Aβ [207]. Moreover, astrocytes express iNOS when challenged with Aβ [208]. ROS and NO production contribute to nitro-oxidative damage in surrounding neurons, with the consequences explained in Section 4 and Section 5 of this review. On the other hand, oxidative stress can impair the function of astrocytes in the maintenance of the BBB [209].

Regarding inflammation, signaling pathways involving nuclear factor-kappa B (NF-κB) and MAPKs are upregulated in reactive astrocytes [210,211], leading to increased production of inflammatory mediators, such as tumor necrosis factor-alpha (TNF-α), IL-1β, and IL-6 [212,213], which are commonly observed in the brains of AD patients. These cytokines contribute to inflammation by promoting the activation of immune cells and the release of additional inflammatory factors. They also induce the release of chemokines [214,215], such as monocyte chemoattractant protein-1 (MCP-1), which play a key role in recruiting immune cells to the site of inflammation [215]. In AD, increased levels of MCP-1 are associated with enhanced microglial activation and inflammation [216]. In summary, proinflammatory mediators and chemokines contribute to an inflammatory environment that exacerbates neuronal damage. Therefore, although initially protective because they phagocytize and degrade Aβ [205,217], reactive astrogliosis becomes detrimental in a vicious cycle of inflammation and oxidative damage.

### 6.2. Microglia in AD

Microglia are the resident immune cells of the brain [218]. Microglia are highly plastic and can adopt different functional states based on environmental signals. Traditionally classified into two very different phenotypes, called M1 (proinflammatory) and M2 (anti-inflammatory), more recent research has stated the complexity of microglial protein expression and behavior, establishing the need to use more detailed non-polarizing nomenclature [219].

#### 6.2.1. Microglia Functions

Upon activation, microglia adopt an amoeboid shape and engage in various functional responses, including phagocytosis, cytokine release, and ROS production [220].

Moreover, microglia carry out the pruning of the synapses during development and in the adult brain [221,222], which is crucial for synaptic plasticity and function [223]. Microglia also support synaptic health by clearing debris [224] and secreting neurotrophic factors, such as brain-derived neurotrophic factor (BDNF) [225] and insulin-like growth factor 1 (IGF1) [226].

#### 6.2.2. Aβ and Microglia

Aβ interacts with pattern recognition receptors (PRRs) on microglia [227], such as toll-like receptors (TLRs) [228], a scavenger receptor [229], and the receptor for advanced glycation end products (RAGE) [230]. All of these facilitate Aβ internalization and degradation [231]. Moreover, microglia secrete neprilysin and insulin-degrading enzyme (IDE) to break down Aβ [232,233]. However, if the Aβ load is too large, another scenario may arise. The binding of Aβ to these PRRs also activates the NADPH oxidase of the membrane, generating a respiratory burst [234], which is a significant source of ROS, with pathophysiological consequences already described in Section 4 and Section 5 of this review. Furthermore, oxidative stress impairs microglial phagocytic function, reducing their ability to clear Aβ plaques and cellular debris [235]. Once NADPH oxidase is activated, microglia produce proinflammatory cytokines (IL-1β, IL-6, TNF-α) and chemokines [236,237]. All these effects contribute to the accumulation of toxic substances in the brain and promote Aβ aggregation and deposition. Microglia can also produce factors that attract more immune cells, creating a noxious feedback loop of inflammation and neurodegeneration, characterized by increased expression of markers such as Iba1 [238].

Astrocytes and microglia closely interact in both physiological and pathophysiological contexts, influencing each other through the release of glutamate, gliotransmitters, cytokines (TNFα, IL1, and IL6 working as paracrine factors), ATP, NO, and ROS [239]. This interaction can either amplify or mitigate the inflammatory response, depending on the surrounding environment. However, it typically becomes harmful once Aβ deposits appear [240].

### 6.3. Inflammation and Mitochondria in AD

Microglial and astrocytic cytokines can impair mitochondrial function by disrupting calcium homeostasis, inhibiting mitochondrial respiration, and promoting ROS production [241]. Furthermore, the transcription factor NF-κB is activated by inflammatory cytokines and can induce the expression of genes that promote oxidative stress and mitochondrial dysfunction [242]. Besides NF-κB, inflammation-induced activation of p53 can lead to transcriptional changes that promote apoptosis and mitochondrial dysfunction [243]. Another classical proinflammatory enzyme, the cyclooxygenase-2 (COX-2), increases its expression in response to inflammation and enhances the production of proinflammatory prostaglandins, exacerbating mitochondrial damage and neuronal death [244].

### 6.4. Specialized Pro-Resolving Mediators

Specialized pro-resolving mediators (SPMs) play a crucial role in resolving inflammation and promoting tissue repair [245,246], and their potential in AD is garnering significant interest [247]. SPMs, including lipoxins, resolvins, protectins, and maresins, are bioactive lipid compounds derived from polyunsaturated fatty acids [246]. In the context of AD, SPMs may help counteract chronic inflammation, a hallmark of the disease, by actively resolving inflammation and facilitating the clearance of Aβ and damaged cells. Emerging research suggests that enhancing SPM pathways could mitigate the progression of AD by restoring homeostasis and protecting neuronal function [248].

### 6.5. Genetic Links to Inflammation in AD

Genetic studies have further evidenced the role of inflammation in AD. Genome-wide association studies (GWAS) have identified several risk genes associated with immune function and inflammation (Supplementary Tables S1 and S2 [249,250,251,252,253,254,255,256,257,258,259,260,261,262,263,264]).

The apolipoprotein E (ApoE) ε4 allele is the strongest genetic risk factor for late-onset AD. ApoE influences Aβ clearance and neuroinflammation, and the ε4 variant is associated with increased Aβ deposition, impaired Aβ clearance, and a heightened inflammatory response [265]. Microglial ApoE ε4 is a disease-associated microglia (DAM) marker, driving immunometabolic changes across the microglial transcriptome, associating with Aβ-independent tau accumulation [266].

Triggering receptor expressed on myeloid cells 2 (TREM2) is crucial for microglial function, including activation, survival, and phagocytosis [267]. TREM2 enhances microglial phagocytosis of Aβ and tau, promotes an anti-inflammatory response to Aβ, and supports lipid metabolism in microglia [267]. TREM2 polymorphisms associated with AD produce a loss of function that increases disease risk by impairing the microglial response to Aβ and tau [257,268,269], leading to increased plaque burden and neuroinflammation. There is a demonstrated interaction between ApoE and TREM2 [270,271]. ApoE seems to facilitate the phagocytosis of dying neurons by activating the TREM2 pathway. However, the R47H variant of TREM2 has been found to lower its binding affinity for ApoE [272]. Moreover, microglial Aβ uptake is enhanced when bound to ApoE, and TREM2-defficient microglia show a reduction in Aβ-ApoE absorption [270]. Although crucial for Aβ clearance, TREM2/ApoE interaction seems to not be essential for phagocytic clearance of dying neural cells, but, interestingly, microglia that lack TREM2 prioritize the phagocytosis of dead cells over Aβ plaques [273].

Complement receptor 1 (CR1) regulates the complement system [274], which is part of the innate immune response. It helps clear immune complexes and cellular debris [275]. In AD, CR1 facilitates Aβ clearance by microglia [276], but genetic variants associated with AD may impair this process [251], leading to increased plaque accumulation and neuroinflammation. CR1 is also involved in synaptic pruning [277], and dysregulation can contribute to synaptic loss and cognitive decline.

CD33/Siglec-3 is an inhibitory receptor on microglia that modulates their immune responses [278]. According to GWAS, CD33 is among the leading genes linked to the risk of developing AD [263]. AD brains show higher levels of CD33 on their microglia, and CD33 presence is correlated with plaque burden and cognitive decline [279,280]. However, CD33 enrichment could be perfectly explained as a result of the neuroinflammation process on those brains. Variants of CD33 that reduce its expression are associated with a lower AD risk [281], as decreased CD33 enhances Aβ clearance and is protective against AD. Conversely, other variants that increase CD33 activity and increase Aβ_1-42_ phagocytosis have recently been correlated with a decreased risk for AD [282]. An explanation to this has been described: the full human CD33 isoform (hCD33M) is correlated with low microglial Aβ clearance, and the isoform resulting from exon 2 exclusion through splicing is correlated with increased phagocytosis (hCD33m) [280,282,283]. Therefore, SNPs that promote either a lower expression of the hCD33M isoform or an enhanced alternative splicing of the protein confer protection against AD.

## 7. The BBB in AD

The BBB is a selective permeability barrier formed by endothelial cells lining the brain’s blood vessels, supported by astrocytic end-feet, pericytes, and a basal lamina [284,285]. The functions of the BBB include: (i) selective permeability by which it regulates the passage of substances between the blood and the brain, allowing essential nutrients to enter while preventing harmful substances from crossing, being able to exclude up to 98% of bloodstream molecules [286]; (ii) brain protection from toxins, pathogens, and fluctuations in blood composition [287], maintaining a stable environment for neuronal function; (iii) maintenance of the extracellular environment, including ion balance and neurotransmitter levels, essential for proper neuronal activity [196].

These singular capacities are related to the presence of tight junctions between the endothelial cells, reducing BBB permeability by limiting all transport through cells except non-ionic molecules [288].

In physiological states, the BBB extrudes Aβ as commented in Section 2. Moreover, it has been demonstrated certain codependence with classical P-Glycoprotein (P-gP), a transporter located in the luminal area of the blood vessel, which mediates Aβ efflux [289,290]. In fact, there is a correlation between LRP-1 and P-gP expression profiles in AD [291,292].

AD is characterized by oxidative stress and inflammation that significantly impact the integrity and function of BBB, especially due to the presence of amyloid deposits surrounding brain vessels, which derive in cerebral amyloid angiopathy. Aβ and ROS damage endothelial cells, inducing apoptosis [164,179]. The rupture of the BBB in AD is a pathological event that results in increased presence of albumin in CSF of AD [293] or the increased Braak stage-dependent presence of prothrombin surrounding capillaries and immunoreactive glia in AD patients [294]. Yamazaki et al. [295] compared the BBB integrity between AD patients, individuals with pathological aging and individuals with normal aging, in 12 different brain areas. AD patients showed a reduction in expression of the tight junction proteins claudin-5 and occludin that correlated with the presence of Aβ1-40 aggregates. These findings may be a consequence of ROS activating various signaling pathways via RhoA, PI3K, and PKB, causing a rearrangement of the actin and a reduction of occluding and claudin-5 [296]. Also, expression of LRP-1 is reduced in endothelial and pericytes in AD, while glia and neurons show increased expression. These results suggest a phenotype change from vascular transcytosis to amyloid phagocytosis [297,298], leading to increased permeability and dysfunction of the tight junction [299]. Moreover, ONOO^−^ can damage endothelial cells by nitrotyrosinating their proteins [164,179], contributing to increased permeability and dysfunction of the BBB.

Inflammation compromises the integrity of the BBB, allowing peripheral inflammatory cytokines and toxic substances to enter the CNS [300]. This exacerbates neuronal injury and inflammation. Astrocytes play a key role in maintaining the integrity of the BBB because they release factors that support endothelial cell tight junctions [301,302], which are also crucial for maintaining the BBB’s integrity. Therefore, astrocyte activation by Aβ impairs their ability the maintain a functional BBB. In fact, the release of TNF-α and IL-1β can disrupt tight junctions and increase BBB permeability [303]. Furthermore, inflammatory mediators can activate matrix metalloproteinases (MMPs) that degrade extracellular matrix components, leading to BBB breakdown [304].

## 8. Oligodendrocytes in AD

While much of the focus in AD research has been on neurons, emerging evidence highlights the significant role of oligodendrocytes, the glial cells responsible for myelinating axons in the brain and supporting neuronal function. Oligodendrocytes are critically affected by oxidative stress and inflammation [305], which collectively contribute to the pathogenesis of AD.

Oligodendrocytes produce myelin, a lipid-rich sheath that insulates axons, facilitating rapid and efficient electrical signal transmission [306], provide metabolic support to neurons by supplying energy substrates and maintaining ionic balance [307], and secrete factors that modulate the inflammatory response and maintain the balance between proinflammatory and anti-inflammatory signals [308].

In AD, oligodendrocytes undergo several pathological changes. Demyelination is a hallmark of AD [309,310,311,312], leading to impaired axonal conduction and cognitive decline. Dysfunctional oligodendrocytes also decrease their neuronal support functions [313]. Activated oligodendrocytes can exacerbate neuroinflammation and contribute to disease progression [314]. Recently, using single-cell RNA sequencing analysis, it has been identified a subpopulation of oligodendrocytes associated with the progression of the disease in both APP^NL-G-F^ and 5xFAD male mice and in AD human brains. They presented an altered Erk1/2 signaling that, when inhibited, rescued impaired axonal myelination and other pathologies [315].

ROS and RNS damage myelin and myelin-producing oligodendrocytes [316], impair the ability of oligodendrocytes to repair damaged myelin [316], and even induce apoptosis in these cells [317,318], impairing axonal conduction and exacerbating disease progression.

Inflammation affects oligodendrocytes since their functions are disrupted by TNF-α and IL-1β [319]. The activation of MMPs contributes to degrading extracellular matrix components of the oligodendrocytes [320], and chronic inflammation impairs the ability of oligodendrocytes to support neuronal function and repair myelin [321,322].

Interestingly, a recent study has pointed out that oligodendrocytes functionally express BACE1, further contributing to the Aβ plaque formation in AD [323].

## 9. Therapeutic Approaches

There is a lot of literature on the mechanisms that contribute to the onset and development of the disease, but there are no specific treatments further than anti-acetylcholinesterase drugs that ameliorate the cholinergic deficit of AD [324]. Memantine is an inhibitor of the glutamate receptors, and clinical studies have shown that it can provide modest improvements in cognitive function but, when combined with anticholinesterasic drugs, may offer additional benefits and improve overall treatment outcomes [325,326]. In addition, more than 200 clinical trials targeting Aβ production have been run, and none was sufficient to recover from AD, and even some of them showed adverse effects [327].

Antibodies are recognized for dismantling Aβ aggregates into monomeric forms through interaction with their Fab regions [328]. Phagocytic cells, such as microglia, express FcγR receptors on their surface for the Fc region of antibodies, contributing to the elimination of Aβ deposits via phagocytosis. Recently, satisfactory results have been obtained with passive immunization with monoclonal antibodies [329,330].

Other therapeutic approaches have probably failed because patients were in advanced stages of the disease, making it hard to get protective results. For this reason, it is essential to diagnose individuals who are in the early asymptomatic phases to start treatment at that time and avoid further complications.

Antioxidant therapies aim to counteract oxidative stress in AD. Agents such as vitamin E [331,332,333,334], coenzyme Q10 [335], and curcumin [336] have been tested in clinical trials with mixed results, indicating a need for further research. Interestingly, amine oxidases, such as monoamine oxidase B (MAO B) and semicarbazide-sensitive amine oxidase (SSAO), play a pathological role in AD by contributing to oxidative stress and neuroinflammation [337,338]. MAO inhibitors, including tranylcypromine, naphthoquinones, and anthraquinone, have been reported to reduce Aβ-induced toxicity by minimizing oxidative stress and even reducing Aβ aggregation [339,340]. The role of MAO inhibitors as anti-inflammatory drugs is also a relevant therapeutic approach for AD [341]. In addition, other anti-inflammatory drugs, including nonsteroidal anti-inflammatory drugs (NSAIDs) [342], cytokine inhibitors [343], and selective COX-2 inhibitors [344,345,346], are being explored for AD treatment. However, their efficacy in altering AD progression remains uncertain, requiring more research.

Adopting a diet rich in antioxidants, like the Mediterranean diet, along with regular exercise and cognitive stimulation, may help reduce oxidative stress and support brain health. It is important to emphasize the publication of data that report a significant decrease in the prevalence of AD in Western countries [347]. This decline is attributed to better control of cardiovascular health and a higher educational level of the population, since it is known that cognitive reserve is a protective factor against AD [348]. Therefore, promotion of good nutritional habits, sport practice [349], and having an acceptable educational level [350] would be a first step in AD prevention until drug therapies become available.

## 10. Conclusions

AD is a multifactorial condition in which Aβ plays a pivotal role. Effective treatment strategies depend on early and accurate diagnosis, especially during the prodromal stages. Current advancements in technology enable the analysis of numerous variables, potentially leading to complex yet precise diagnostic systems in the near future.

The future of AD treatments should focus on three key areas: first, inhibiting Aβ production or reducing its levels, acknowledging its potential physiological roles; second, preventing Aβ aggregation during the early prodromal phases is crucial since dismantling existing aggregates can lead to an uncontrolled rise in soluble Aβ, which may migrate to blood vessels, causing cerebral amyloid angiopathy; and third, protecting against inflammation and neurotoxicity is essential, and the use of antioxidants and anti-inflammatory drugs should continue to be tested.

In conclusion, a comprehensive approach targeting Aβ production, aggregation, and the associated oxidative stress and inflammation is critical for advancing AD treatment. Future research should focus on early detection and multifaceted intervention strategies to slow or halt disease progression and improve patient outcomes.

## Data Availability

Not applicable.

## Supplementary Materials

The following supporting information can be downloaded at: https://www.mdpi.com/article/10.3390/antiox13101208/s1, Table S1: Polymorphisms that contribute to Alzheimer’s disease onset and progression; Table S2: Polymorphisms identified in a GWAS performed with a South Brazilian population.

## Abbreviations

4-HNE, 4-Hydroxynonenal; 8-NO2G, 8-Nitroguanine; 8-OHdG, 8-Hydroxy-2′-Deoxyguanosine; ACE, angiotensin-converting enzyme; AD, Alzheimer’s disease; AGEs, advanced glycation end-products; AICD, amyloid precursor protein intracellular domain; APH1, anterior pharynx defective 1; APLP, APP-like proteins; ApoE, apolipoprotein E; APP, amyloid precursor protein; Aβ, amyloid beta-peptide; BACE1, β-site APP cleaving enzyme 1; BBB, blood–brain barrier; BDNF, brain-derived neurotrophic factor; CaMKIIα, calcium/calmodulin-dependent protein kinase II alpha; CNS, central nervous system; COX-2, cyclooxygenase-2; CR1, complement receptor 1; CREB, cAMP response element-binding protein; CTF, C-terminal fragment; DMS-5, Diagnostic and Statistical Manual of Mental Disorders; ECE, endothelin-converting enzyme; eIF2-alpha, eukaryotic translation initiation factor 2-alpha; eNOS, endothelial nitric oxide synthase; ETC, electron transport chain; Fab, fragment antigen-binding; Fc, fragment crystallizable; GFAP, glial fibrillary acidic protein; GLT-1, glutamate transporter 1; GSH, glutathione; GSK-3β, glycogen synthase kinase 3 beta; GWAS, genome-wide association studies; HSV-1, herpes simplex virus 1; IDE, insulin-degrading enzyme; IGF1, insulin-like growth factor 1; IL, interleukin; JNK, c-Jun N-terminal kinase; LRP1, low-density lipoprotein receptor-related protein 1; LTP, long-term potentiation; MCP-1, monocyte chemoattractant protein-1; MDA, malondialdehyde; MMPs, matrix metalloproteinases; mtDNA, mitochondrial DNA; mtNOS, mitochondrial nitric oxide synthase; NCT, nicastrin; NEP, neprilysin; NF-κB, nuclear factor-kappa B; NICD, notch intracellular domain; NMDAR, N-methyl-D-aspartate receptor; nNOS, neuronal nitric oxide synthase; NO, nitric oxide; NSAIDs, nonsteroidal anti-inflammatory drugs; NTF, N-terminal fragment; ONOO-, Peroxynitrite; p38-MAPK, p38 mitogen-activated protein kinase; PEN-2, presenilin enhancer 2; PET, positron emission tomography; PHFs, paired helical filaments; PRRs, pattern recognition receptors; PSEN, presenilin; RAGE, receptor for advanced glycation end products; RNS, reactive nitrogen species; ROS, reactive oxygen species; RyR, ryanodine receptor; sAPPβ, soluble amyloid precursor protein-beta; SPMs, specialized pro-resolving mediators; TLRs, toll-like receptors; TNF-α, tumor necrosis factor-alpha; TREM2, triggering receptor expressed on myeloid cells 2; VDAC, voltage-dependent anion channel; VLDLR, very low-density lipoprotein receptor.
