# Supplementary material for: The Dual Role of Amyloid Beta-Peptide in Oxidative Stress and Inflammation: Unveiling Their Connections in Alzheimer’s Disease Etiopathology
Source: Antioxidants (Basel). 2024 Oct 8;13(10):1208. doi: 10.3390/antiox13101208 (PMC11505517; doi:10.3390/antiox13101208)
Supplement: Supplementary file 1 [file antioxidants-13-01208-s001.zip › antioxidants-3172111-supplementary.pdf]

SUPPLEMENTARY MATERIAL

Table S1. Polymorphisms that contribute to Alzheimer's disease onset and progression

SNP	Closest gene(s)	Protein	Protein function	Reference
rs429358	*APOE*	ApoE	Apolipoprotein of the chylomicron. It binds to a specific liver and peripheral cell receptor, and is essential for the normal catabolism of triglyceride-rich lipoprotein constituents. The major genetic risk factor for LOAD.	[249,250]
rs6656401	*CR1*	CR1	Member of the receptors of complement activation (RCA) family. Mediates cellular binding to particles and immune complexes that have activated complement.	[251]
rs6733839	*BIN1*	BIN1	Nucleocytoplasmic adaptor protein. Isoforms that are expressed in the central nervous system may be involved in synaptic vesicle endocytosis and may interact with dynamin, synaptojanin, endophilin, and clathrin.	[250,252]
rs10948363	*CD2AP*	CD2AP	Scaffolding molecule that regulates the actin cytoskeleton. Implicated in dynamic actin remodeling and membrane trafficking that occurs during receptor endocytosis and cytokinesis.	[253,254]
rs11771145	*EPHA1*	EPHA1	Upon activation by EFNA1 induces cell attachment to the extracellular matrix inhibiting cell spreading and motility through regulation of ILK and downstream RHOA and RAC. Also plays a role in angiogenesis and regulates cell proliferation. May play a role in apoptosis.	[253,254]
rs9331896	*CLU*	CLU	Extracellular chaperone that prevents aggregation of non-native proteins. Prevents stress-induced aggregation of blood plasma proteins. Inhibits formation of amyloid fibrils by APP, APOC2, B2M, CALCA, CSN3, SNCA.	[251,252,255]
rs983392	*MS4A6A/E*	MS4A6A/E	May be involved in signal transduction as a component of a multimeric receptor complex.	[253,254]
rs10792832	*PICALM*	PICALM	Recruits clathrin and adaptor protein complex 2 (AP2) to cell membranes at sites of coated-pit formation and clathrin-vesicle assembly. The protein may be required to determine the amount of membrane to be recycled, possibly by regulating the size of the clathrin cage.	[252,255]
rs4147929	*ABCA7*	ABCA7	ATP-binding cassette transporter detected predominantly in myelo-lymphatic tissues. May have a role in lipid homeostasis in cells of the immune system.	[254]
rs3865444	*CD33*	CD33	Enables protein phosphatase binding activity and sialic acid binding activity. Involved in several processes, including negative regulation of cytokine production; negative regulation of monocyte activation; and positive regulation of protein tyrosine phosphatase activity.	[253,254]

rs2974151	*CTNNA2*	CTNNA2 (Amish)	Enables actin filament binding activity. Involved in negative regulation of Arp2/3 complex-mediated actin nucleation; regulation of neuron migration; and regulation of neuron projection development.	[256]
rs75932628-T	*TREM2*	TREM2	The encoded protein functions in immune response and may be involved in chronic inflammation by triggering the production of constitutive inflammatory cytokines. Present in the microglia.	[257]
rs9271192	*HLA-DRB5 and HLA-DRB1*	HLA-DRB5 and HLA-DRB1	Play a central role in the immune system by presenting peptides derived from extracellular proteins. Expressed in antigen presenting cells.	[258]
rs28834970	*PTK2B*	PTK2B	Tyrosine kinase involved in calcium-induced regulation of ion channels and activation of the map kinase signaling pathway. May represent an important signaling intermediate between neuropeptide-activated receptors or neurotransmitters that increase calcium flux and the downstream signals that regulate neuronal activity.	[258]
rs11218343	*SORL1*	SORL1	Mosaic protein that belongs to at least two families: the vacuolar protein sorting 10 (VPS10) domain-containing receptor family, and the low-density lipoprotein receptor (LDLR) family.	[258]
rs10498633	*SLC24A4 and RIN3*	SLC24A4 and RIN3	SLC24A4: member of the potassium-dependent sodium/calcium exchanger protein family. Controls the rapid response termination and proper regulation of adaptation in olfactory sensory neurons.<br>RIN3: Ras effector protein that functions as a guanine nucleotide exchange (GEF) for RAB5B and RAB31, by exchanging bound GDP for free GTP. Required for normal RAB31 function.	[258]
rs8093731	*DSG2*	DSG2	Calcium-binding transmembrane glycoprotein component of desmosomes, cell-cell junctions between epithelial, myocardial, and other cell types.	[258]
rs35349669	*INPP5D*	INPP5D	Hydrolyzes the 5' phosphate from phosphatidylinositol (3,4,5)-trisphosphate and inositol-1,3,4,5-tetrakisphosphate, thereby affecting multiple signaling pathways. Functions as a negative regulator of myeloid cell proliferation and survival.	[258]
rs190982	*MEF2C*	MEF2C	Plays a role in myogenesis. Has both trans-activating and DNA binding activities. This protein may play a role in maintaining the differentiated state of muscle cells.	[258]
rs2718058	*NME8*	NME8	Probably required during the final stages of sperm tail maturation in the testis and/or epididymis, where extensive disulfide bonding of fibrous sheath (FS) proteins occurs. May be involved in the reduction of disulfide bonds within the sperm FS components.	[258]
rs1476679	*ZCWPW1*	ZCWPW1	Dual histone methylation reader specific for PRDM9-catalyzed histone marks (H3K4me3 and H3K36me3). Facilitates the repair of PRDM9-induced meiotic double-strand breaks (DSBs) (By	[258]

			similarity). Essential for male fertility and spermatogenesis	
rs10838725	*CELF1*	CELF1	RNA-binding protein implicated in the regulation of several post-transcriptional events. Involved in pre-mRNA alternative splicing, mRNA translation and stability. Inhibits, together with HNRNPH1, insulin receptor (IR) pre-mRNA exon 11 inclusion in myoblast.	[250,258]
rs17125944	*FERMT2*	FERMT2	Scaffolding protein that enhances integrin activation. Participates in the connection between extracellular matrix adhesion sites and the actin cytoskeleton, and also in the orchestration of actin assembly and cell shape modulation.	[258]
rs7274581	*CASS4*	CASS4	Enables protein tyrosine kinase binding activity. Involved in several processes, including positive regulation of protein kinase B signaling; positive regulation of protein tyrosine kinase activity; and positive regulation of substrate adhesion-dependent cell spreading.	[258]
rs112404845	*COBL*	COBL	Important role in the reorganization of the actin cytoskeleton. Regulates neuron morphogenesis and increases branching of axons and dendrites. Regulates dendrite branching in Purkinje cells.	[259]
rs16961023	*SLC10A2*	SLC10A2	Sodium/bile acid cotransporter. This transporter is the primary mechanism for uptake of intestinal bile acids by apical cells in the distal ileum.	[259]
rs616338	*ABI3*	ABI3	May inhibit tumor metastasis. In vitro, reduces cell motility.	[260]
rs72824905	*PLCG2*	PLCG2	Catalyzes the conversion of 1-phosphatidyl-1D-myo-inositol 4,5-bisphosphate to 1D-myo-inositol 1,4,5-trisphosphate (IP3) and diacylglycerol (DAG) using calcium as a cofactor.	[260]
rs10984186	*CDK5RAP2*	CDK5RAP2	Potential regulator of CDK5 activity via its interaction with CDK5R1. Negative regulator of centriole disengagement, which maintains centriole engagement and cohesion. Involved in regulation of mitotic spindle orientation.	[261]
rs4735340	*NDUFAF6*	NDUFAF6	Assembly of complex I (NADH-ubiquinone oxidoreductase) of the mitochondrial respiratory chain through regulation of subunit ND1 biogenesis.	[262]
rs7920721	*ECHDC3*	ECHDC3	Predicted to enable enoyl-CoA hydratase activity. Involved in positive regulation of cellular response to insulin stimulus.	[262]
rs7295246	*ADAMTS20*	ADAMTS20	Member of the ADAMTS family of zinc-dependent proteases. May be involved in tissue remodeling.	[262]
rs10467994	*SPPL2A*	SPPL2A	Member of the GXGD family of aspartic proteases that localizes to late endosomal compartments and lysosomal membranes.	[262]
rs593742	*ADAM10*	ADAM10	Metalloprotease that cleaves many proteins including TNF-α, E-cadherin and APP.	[262]
rs7185636	*IQCK*	IQCK	Presents an IQ-motif that serves as a binding site for different EF-hand proteins such as	[262]

			calmodulin. Linked to obsessive-compulsive disorder.	
rs2632516	*MIR142 and TSPOAP1-AS1d*	MIR142 (miRNA) and TSPOAP1-AS1d	MIR142: belongs to the miRNA class. Associated to brain cancer and multiple sclerosis.<br><br>TSPOAP1- AS1d: enables benzodiazepine receptor binding activity. Predicted to be involved in regulation of presynaptic cytosolic calcium ion concentration	[262]
rs138190086	*ACE*	ACE	Involved in blood pressure regulation and electrolyte balance. It catalyzes the conversion of angiotensin I into a physiologically active peptide angiotensin II. Also implicated in Aβ clearance.	[262]
rs2830500	*ADAMTS1*	ADAMTS1	Presents anti-angiogenic activity. Associated with various inflammatory processes as well as development of cancer cachexia. This gene is likely to be necessary for normal growth, fertility, and organ morphology and function.	[262]
rs71618613	*SUCLG2P4*	SUCLG2P4	Pseudogene 4 of SUCLG2, a GTP-specific beta subunit of succinyl-CoA synthetase. Succinyl-CoA synthetase catalyzes the reversible reaction involving the formation of succinyl-CoA and succinate.	[262]
rs35868327	*FST T/A*	FST T/A	Gonadal protein that specifically inhibits follicle-stimulating hormone release. Linked to polycystic ovary syndrome.	[262]
rs114812713	*OARD1*	OARD1	Can act as a glutamate mono-ADP-ribosylhydrolase. Linked to renal dysfunction.	[262]
rs62039712	*WWOX*	WWOX	Putative oxidoreductase. Acts as a tumor suppressor and plays a role in apoptosis.	[262]
rs113020870	*AGRN*	AGRN	Critical in the development of the neuromuscular junction.	[263]
rs871269	*TNIP1*	TNIP1	Role in autoimmunity and tissue homeostasis through the regulation of nuclear factor kappa-B activation.	[263]
rs6891966	*HAVCR2*	HAVCR2	Th1-specific cell surface protein that regulates macrophage activation, and inhibits Th1-mediated auto- and alloimmune responses, and promotes immunological tolerance.	[263]
rs5011436	*TMEM106B*	TMEM106B	Enables ATPase binding activity. Involved in dendrite morphogenesis and lysosome localization. Located in endosome and lysosomal membrane.	[263]
rs708382	*GRN*	GRN	Secreted protein that acts as a key regulator of lysosomal function and as a growth factor involved in inflammation, wound healing and cell proliferation.	[263]
rs2452170	*NTN5*	NTN5	Predicted to enable signaling receptor binding activity. Predicted to be involved in animal organ morphogenesis; neuron projection development; and tissue development.	[263]
rs1761461	*LILRB2*	LILRB2	Expressed on immune cells where it binds to MHC class I molecules on antigen-presenting cells and transduces a negative signal that	[263]

			inhibits stimulation of an immune response. It is thought to control inflammatory responses and cytotoxicity to help focus the immune response and limit autoreactivity.	
rs189753894	*CACNA1A*	CACNA1A	Mediates the entry of calcium ions into excitable cells, and are also involved in a variety of calcium-dependent processes, including muscle contraction, hormone or neurotransmitter release, and gene expression.	[264]
rs2280575	*LRIG1*	LRIG1	Predicted to act upstream of or within several processes, including innervation; otolith morphogenesis; and sensory perception of sound.	[264]
rs10119	*TOMM40*	TOMM40	Channel-forming protein essential for import of protein precursors into mitochondria. Plays a role in the assembly of the mitochondrial membrane respiratory chain NADH dehydrogenase (Complex I).	[264]
rs12972156	*PVRL2*	PVRL2	Modulator of T-cell signaling. Can be either a costimulator of T-cell function, or a coinhibitor, depending on the receptor it binds to. Upon binding to CD226, stimulates T-cell proliferation and cytokine production.	[264]

Polymorphisms identified in genome-wide association studies (GWAS) regarding late-onset Alzheimer's disease from GWAS Catalog and in previous studies. The studies include Asian, African-American, Brazilian, Amish and European populations.

Table S2. Polymorphisms identified in a GWAS performed with a South Brazilian population

SNP	Long non-coding RNA ID	Reference
rs71457224<br>rs10769282	NONHSAT021264.2	[250]
rs10414043	NONHSAT179794.1	[250]
rs7256200<br>rs429358	NONHSAT066732.2	[250]
rs429358	NONHSAT179793.1	[250]
rs4663105	NONHSAT187478.1	[250]
rs744373<br>rs730482	NONHSAT182593.1	[250]

The closest gene to the polymorphisms does not encode for a protein but for a long non-coding RNA.
